# Genetic alterations and field defect in head and neck squamous cell carcinoma

**DOI:** 10.1186/1897-4287-10-S4-A21

**Published:** 2012-12-10

**Authors:** J K Strzelczyk, Ł Krakowczyk, M Kwaśniewski, A Płachetka, K Gołąbek, K Chwiałkowska, A Maciejewski, A Wiczkowski

**Affiliations:** 1 Chair and Department of General Biology, Medical University of Silesia, Jordana 19 Str., 41-808 Zabrze, Poland; 2 Clinic of Oncological and Reconstructive Surgery, Maria Sklodowska–Curie Memorial Cancer Center and Institute of Oncology, Gliwice Branch, Wybrzeże Armii Krajowej 15 Str., 44-101 Gliwice, Poland; 3 Department of Genetics University of Silesia Jagiellońska 28 40-032 Katowice, Poland

## 

A genetic progression model of head and neck squamous cell carcinoma (HNSCC) has not yet been elucidated, and the genetic basis for "field cancerization" has also remained unclear. Most of the "field cancerization" has been explained by the presence of cells with genetic alterations, however, involvement of epigenetic alterations in field cancerization was shown, too.

In the present study, paired specimens of tumour and adjacent normal tissues were obtained from materials surgically resected from 25 patients with squamous cell carcinoma of the head and neck. After extraction of RNA, quantitative RT-PCR method using 7300 TaqMan (AppliedBiosystems) was used to analyze gene expression of *MGMT*, *p16*, *TIMP3.* Next, we examined the association between *MGMT*, *p16*, *TIMP3* promoters methylation and genes expression. The studies demonstrated higher expression of *p16* gene in tumour compared with adjacent normal tissues. Other, *MGMT* and *TIMP3* showed no differences. Our results revealed no correlation between *MGMT*, *p16*, *TIMP3* promoters methylation level and expression of these genes. Finally, we performed direct DNA sequence analysis of *MGMT* somatic mutations both in tumour and adjacent normal tissue. *MGMT* is a 300,000 bp-long gene that consist of six relatively short exons. The gene is transcribed as a 1265 bp-long mRNA, covering the 717 bp-long coding sequence. To facilitate the screening of somatic mutations in the coding region of the *MGMT*, a simple approach has been developed. As the gene is expressed in HNSCC, we decided to amplify the whole coding region of *MGMT* in two separate PCR reactions using its cDNA as a template. The amplicons of individual patients were labeled in PCR with MID identifiers and sequenced using a medium-scale next generation sequencing system, GS Junior (Roche/454). The method developed can accurately identify low-level mutations, down to a level of 5% of cells within the testing sample.

The lack of progress in head and neck oncology emphasizes the importance of molecular genetic studies to define alterations that may correlate with tumor behavior. Further studies may help clarify this issue.

